# Editorial: Molecular Nanomachines of the Presynaptic Terminal

**DOI:** 10.3389/fnsyn.2016.00027

**Published:** 2016-08-25

**Authors:** Silvio O. Rizzoli, Lucia Tabares

**Affiliations:** ^1^Department of Neuro- and Sensory Physiology, University Medical Center GöttingenGöttingen, Germany; ^2^Medical Physiology and Biophysics, University of SevilleSeville, Spain

**Keywords:** synapse, neurotransmitter release, active zone, nanomachine, synaptic organization

Cell biology and neurobiology have started many decades ago as attempts to unravel the complexity of cellular processes, by studying different elements, from membranes and organelles to molecules in isolation. Through ever increasing technological improvements, a huge amount of information has accumulated on the molecular organization of the cell, and on the function of the individual molecules. On the basis of this information, we can now switch the general aim of cell biology from the investigation of single elements to the global understanding of cellular function, to a level that will enable us to predict its behavior.

We are not quite there yet, but we are getting close for one compartment of the cell, which is sufficiently well understood at the level of the individual cellular machineries: the synapse, and especially the presynaptic bouton. The bouton is small, contains a limited subset of proteins and organelles, and has a single major function: neurotransmitter release. The molecules and organelles arrange themselves in complexes, or nanomachines, concerned with a handful of critical functional steps, from synaptic vesicle fusion (exocytosis), to vesicle reformation, retrieval (endocytosis), and refilling with neurotransmitter. This Research Topic presents an overview of the most important nanomachines of the presynaptic bouton. Their function and regulation are presented in a level of detail that surpasses that available for most other cellular structures.

Perhaps the most complex, and the most organized machinery of the presynaptic bouton is the active zone, the electron-dense area where synaptic vesicles dock and fuse. This is the subject for two of our chapters, focusing on scaffolding proteins that are essential for this structure (Gundelfinger et al.), and on its plasticity (Kittel and Heckmann). The active zone is probably also linked to the periactive zone, an area where the recently exocytosed synaptic vesicles may prepare for endocytosis. The organization of the periactive zone is far less well understood than that of the active zone, and is here discussed by Cano and Tabares.

The interaction of the active zone with the synaptic vesicles is one of the factors determining the probability with which the latter release neurotransmitter. This is discussed at length by Körber and Kuner. Albeit most of the molecules involved in neurotransmitter release, and especially the fusion SNARE proteins, have been known for several decades, they can still offer surprises, especially when they present function that are not related to exocytosis. This is presented in detail by Michela Matteoli and colleagues, focusing on the most abundant of the SNAREs, SNAP-25 (Antonucci et al.).

Exocytosis needs to be balanced by endocytosis, to ensure the long-term function of the synapses. The molecules involved in endocytosis have been studied for many years, just as the SNAREs, albeit many points are still open. For example, it is still unclear whether synaptic vesicles maintain their composition after exocytosis, as a patch of molecules in the plasma membrane, or whether their components diffuse and intermix with other plasma membrane molecules. The first scenario predicts that one synaptic vesicle will maintain its protein composition, and thereby its identity, for long time periods, not only of a few minutes, but of hours, through many rounds of synaptic vesicle recycling. The second scenario predicts that the synaptic vesicle composition is far more fluid, and will change on a timescale of minutes. The key to this question, which is still open, probably lies within the molecules that interact with the vesicle proteins after exocytosis, and may prevent their diffusion in the plasma membrane. Gordon and Cousin term such molecules intrinsic trafficking partners or iTRAPs. Furthermore, additional nanomachines involved in the fate of the vesicle are discussed by Fassio et al.

After endocytosis, the synaptic vesicle is re-filled with neurotransmitter. This process is far more complex than, for example, SNARE-mediated exocytosis, albeit the regulation of the latter through various proteins, including the active zone components, may be even more complex than neurotransmitter uptake into vesicles. The molecules involved in this process, and their relation to the amount of neurotransmitter loaded into individual vesicles, are presented by Takamori. One hypothesis that was once shunned, but increasingly wins acceptance, is that specific subsets of neurons may contain vesicles loaded with different small transmitters, thereby resulting in more complex signaling than previously thought. A timely review of this subject is given by Ahnert-Hilger et al. (Münster-Wandowski).

None of the synaptic functions discussed so far could be performed without a steady flow of proteins and organelles to and from the synapse, to ensure the replacement of old and damaged components with fresh ones. This subject, which is surprisingly less well-known than many other synaptic aspects, is reviewed by Yagensky et al.

Finally, components of nanomachines can also go wrong, and result in synaptic damage. This is most evident for the amyloid precursor protein, APP, which has been for many years known as the main components of plaques in Alzheimer's Disease. Its physiological and pathophysiological roles are reviewed here by Walter Volknandt and colleagues (Laßek et al.), focusing on the interactions of APP with other nanomachines discussed in this Research Topic.

Overall, this Research Topic therefore provides a timely look at some of the machines composing one of the best-understood compartments of the cell, the synaptic bouton. We hope that the different chapters will spark further quantitative work on the functional assemblies described here, and will also encourage research into nanomachines that are still poorly understood.

## Author contributions

Both authors wrote the manuscript together, and approved it for publication.

### Conflict of interest statement

The authors declare that the research was conducted in the absence of any commercial or financial relationships that could be construed as a potential conflict of interest.

